# 

**Published:** 1884-11

**Authors:** 


### BOOKS AND PAMPHLETS RECEIVED.

*The National Dispensatory*, third edition; Henry C. Lea's Son & Co.; Philadelphia, 1884.

*Medical Record*, Visiting List, or Physician's Diary for 1885. New York: Wm. Wood & Co.

*The Lock Jaw of Infants* (Trismus Nascentium), or Nine-Day Fits, Crying, Spasms, etc. Its History, Cause, Prevention and Cure. By J. F. Hartigan, M.D., Washington, D. C., Member of the American Medical Association, etc. New York: Bermingham & Co., 1884.

*E. Duncan Sniffin*, 3 Park Row, New York; Advertisers' Reference Book, 1884.

*Transactions* of the Lousiana State Medical Society, at its sixth Annual Session, held at Baton Rouge, La., May 21, 22 and 23, 1884; P. B. McCuthon, M. D., Secretary: New Orleans.

*Transactions* of the Medical and Chirurgical Faculty of the State of Maryland; Eighty-sixth Annual Session, held at Baltimore, Md., April 1884; T. Barton Brune, M.D., Secretary, Baltimore.

*On the Development* of Physiological Chemistry and its Significance for Medicine; by Felix Hoppe-Seyler. Translated by T. Wesley Mills, M.A., M.D., Demonstrator of Physiology, McGill University, Montreal, Canada. Reprinted from the New York Medical Journal for August 16 and 23, 1884.

*Diphtheria Spread by Adults;* by A. Jacobi, M.D., Clinical Professor of Diseases of Children in the College of Physicians and Surgeons, New York. Reprinted from the New York Medical Journal for September 27, 1884.

### OUR ADVERTISERS.

*The New York Pharmaceutical Co., Bedford Springs, Mass.*—This company has an attractive advertisement in this issue of THE JOURNAL, to which we desire to direct the attention of our readers.

*Lynch & Lester, Atlanta.*—This old established firm that has been selling books in Atlanta for over thirty years, have recently added to their stock a large supply of medical books of all kinds, which they propose to sell for ten per cent. less than publishers' prices. They will order any medical work which they may not have in stock at the same reduced rate. See their advertisement.

*I. Philips, Atlanta.*—Attention is directed to the advertisement of this gentleman. He deals in instruments and surgical appliances exclusively, and is the only exclusive instrument dealer in the South. At his place may be found any instrument or surgical appliance that physicians may need. Mr. Phillips has been engaged in the instrument business for over twenty years and knows the wants of the profession. Give him a call and examine his stock.

*L. H. Bradfield, Atlanta.*—Nothing is more desirable than that physicians' prescriptions should be filled by a competent prescriptionist with pure and fresh drugs. This can and will always be done by the gentleman whose name appears at the head of this notice. He is one of the oldest druggists in the city and thoroughly understands his business. No prescriptionist in the State stands higher with the profession than does Mr. Bradfield. See his card to be found among the new advertisements in this issue.

*Bill Arp's Scrap-Book.*—No one with a mind and heart to take in true wisdom and a world of sunny humor, should fail to get a copy of this wonderful book. Publisher's price $2.00. It will be sent with THE JOURNAL for $3.50, post-paid.

### CONTENTS.

ORIGINAL COMMUNICATIONS—

### NOTICE.

Original communications solicited. When articles require illustration the engravings will be furnished by the publishers free of charge.

Subscription: $2.50 a year.

Subscribers will please not send us money by Postal Notes. Remit by Express, Draft, Post-office Order, or Registered Letter.

Address all letters and books, and make all drafts payable to

The Atlanta Medical and Surgical Journal,
Care Jas. P. Harrison & Co., Atlanta, Ga.

